# The Tenth Annual Session of the New Jersey State Dental Society

**Published:** 1880-06

**Authors:** Chas. A. Meeker

**Affiliations:** Sec’y, Newark, N. J.


Friend Meeker sends us the following notice:

THE TENTH ANNUAL SESSION OF THE NEW JERSEY STATE DENTAL SOCIETY, will be held at Long Branch, July 20th, 21st and 22d. The Board of Examiners will meet from the 19th, for the examination of candidates for license to practice. The profession generally are invited to be present. Long Branch is easy of access, with unsurpassed hotel accommodations. Every attention will be given to Inventors and Dealers in Dental Appliances, for the proper exhibition of their goods; those so desiring will communicate with Dr. J. C. Clarke, Chairman Executive Committee, Jersey City.

CHAS. A. MEEKER, Sec'y,
Newark, N. J.

